# The Development of an IMU Integrated Clothes for Postural Monitoring Using Conductive Yarn and Interconnecting Technology

**DOI:** 10.3390/s17112560

**Published:** 2017-11-07

**Authors:** Sung-Won Kang, Hyeob Choi, Hyung-Il Park, Byoung-Gun Choi, Hyobin Im, Dongjun Shin, Young-Giu Jung, Jun-Young Lee, Hong-Won Park, Sukyung Park, Jung-Sim Roh

**Affiliations:** 1 Electronics and Telecommunications Research Institute, Daejeon 34129, Korea; kangsw@etri.re.kr (S.-W.K.); hipark@etri.re.kr (H.-I.P.); cbgun@etri.re.kr (B.-G.C.); 2 Department of Mechanical Engineering, Korea Advanced Institute of Science and Technology (KAIST), Daejeon 34141, Korea; hyeobchoi@kaist.ac.kr; 3 Department of Fashion & Textiles, konkuk University, Seoul 05029, Korea; hyobin25@nate.com; 4 BNSoft, Inc., Seoul 08378, Korea; djshin@bns.co.kr; 5 YM-Naeultech, Incheon 22212, Korea; youngq.jung@ym-naeultech.com (Y.-G.J.); jylee@ym-naeultech.com (J.-Y.L.); 6 Korea High Tech Textile Research Institute, Yangju-si 11410, Korea; parkong1@koteri.re.kr; 7 Department of Fashion & Textiles, Sangmyung University, Seoul 03016, Korea

**Keywords:** wearable, posture, monitor, smart wear, IMU modules, conductive yarn

## Abstract

Spinal disease is a common yet important condition that occurs because of inappropriate posture. Prevention could be achieved by continuous posture monitoring, but most measurement systems cannot be used in daily life due to factors such as burdensome wires and large sensing modules. To improve upon these weaknesses, we developed comfortable “smart wear” for posture measurement using conductive yarn for circuit patterning and a flexible printed circuit board (FPCB) for interconnections. The conductive yarn was made by twisting polyester yarn and metal filaments, and the resistance per unit length was about 0.05 Ω/cm. An embroidered circuit was made using the conductive yarn, which showed increased yield strength and uniform electrical resistance per unit length. Circuit networks of sensors and FPCBs for interconnection were integrated into clothes using a computer numerical control (CNC) embroidery process. The system was calibrated and verified by comparing the values measured by the smart wear with those measured by a motion capture camera system. Six subjects performed fixed movements and free computer work, and, with this system, we were able to measure the anterior/posterior direction tilt angle with an error of less than 4°. The smart wear does not have excessive wires, and its structure will be optimized for better posture estimation in a later study.

## 1. Introduction

Wearable systems integrated with clothes have been extensively studied in recent years to monitor body posture without disturbing natural human activity [1,2,3,4,5,6,7,8,9]. Several electro-textile techniques are required to develop “smart wear” for postural data collection and communication with a host computer, such as conductive yarn with high strength and low electrical resistance, stretchable electronic circuitry, and robust fabric–sensor interconnections [10,11,12,13]. A stretchable circuit network made from fabric is recommended for comfortable smart wear to collect motion data and provide real-time feed back to the wearer.

Several studies have developed wearable posture monitoring systems, but most of the systems are difficult to use in daily life. Gopalai and Senanayake developed a system that can measure posture and give feedback by vibrotactile stimulus, but the system was not easy to use due to the bulky sensor module and wires [1]. Several studies have attempted to increase wearability through the development of flexible or light sensors, but they had some limitations such as a variety of measurement data. Sardini et al. developed a fabric sensing system for posture measurement, but it could only be used to measure in one direction and required a large circuit board for the power supply, which was worn on the waist [2]. Mattmann et al. developed textile strain sensors for posture measurement, but it could only measure the bending of the trunk, not the trunk angle [3]. Dunne et al. developed a trunk posture measurement system using an optical sensor. The sensors were small, so the system’s weight was similar to that of daily cloth, but only the spinal angle could be measured [4].

In this study, we developed an integrated smart suit for postural monitoring and performed a feasibility test of the prototype. Custom-made, lightweight, and compact inertial measurement unit (IMU) sensor modules were developed to measure posture. Conductive yarn with high strength and low resistance was developed to establish a stretchable conductive circuit network for sensors. A technique to connect the yarn and sensors was developed to secure the robustness of the smart clothes. The feasibility of the smart wear prototype was tested for its ability to monitor sitting posture.

## 2. Methods

The purpose of this study was to develop a motion sensing system optimized for clothes so that information regarding the user’s daily life motion could be obtained. To this end, (1) an inertial sensor module and monitoring system for smart clothing was developed, (2) to ensure that the entire system was naturally integrated into clothes, conductive-yarn-based circuit patterning technology and interconnecting technology were developed, and (3) the proposed smart clothing was tested for its ability to monitor sitting posture.

### 2.1. Motion Monitoring Process of Smart Clothing

**Figure 1:**
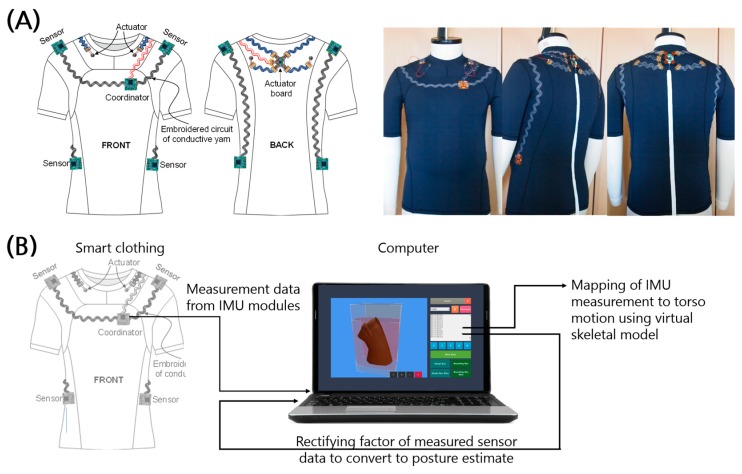
Schematics of IMU-integrated clothes for postural monitoring. (**A**) The sensor–actuator network, the cutting lines of the garment, and a prototype of the smart clothes. (**B**) A block diagram of the process of postural monitoring using the proposed smart clothing.

The proposed smart clothing integrates IMU modules with conductive yarn and fabric-based interconnectors (Figure 1). The overall monitoring process is shown in Figure 1b. First, the postural information is measured with four IMU sensors located at each shoulder and on each side of the waist. The information is integrated and rectified in a processor called a coordinator located 6 cm to the left of the front center and 19 cm below the side of the neck of the garment. The number and location of the sensors were chosen by considering the option of expanding the system to capture whole-body motion in future work. The sensors are located at joints, and the locations were chosen to suppress the effect of moving skin and muscles. Rectified postural information was sent via serial communication to a computer, which maps the sensor information to points on a virtual skeleton and transforms a virtual torso to reflect the measured posture. 

### 2.2. Sensors and Actuators

**Figure 2:**
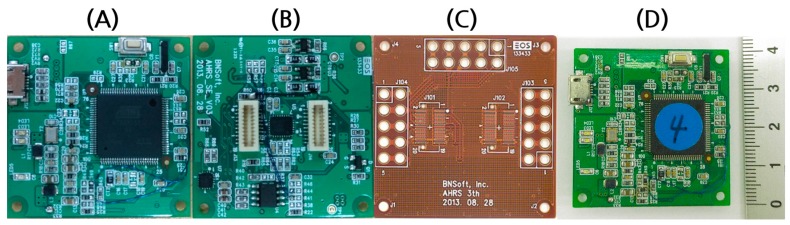
(**A**) Front and (**B**) back views of the custom-made IMU sensor module and (**C**) flexible printed circuit board (FPCB) for interconnection. (**D**) Combined sensor modules attached on the FPCB.

The custom-designed IMU module consists of a 3-axis accelerometer (ADXL346, Analog Device, Norwood, MA, USA), 3-axis gyroscope (L3G4200D, STMicroelectronics, Geneva, Switzerland), and 3-axis magnetometer (HMC5883L, Honeywell, Morris Plains, NJ, USA), all of which are embedded in a microcontroller unit (Cortex SAM3S2C LQPF100, Atmel, San Jose, CA, USA) (Figure 2). We designed this 9-axis sensor module with a magnetometer to remove the drift error in the yaw data and reduce the error in the roll and pitch data. The microcontroller unit estimates the roll, pitch, and yaw using the outputs of the sensors through estimation and prediction based on a Kalman filter. The model is lighter (6 g) and smaller (40 × 40 × 2 mm) than conventional IMU modules used for commercial wearable sensor systems, such as those from APDM (22 g, 48 × 36 × 13 mm) and Shimmer3 (24 g, 51 × 34 × 14 mm). The coverage of each sensor measurement is ±16 g for the accelerometer, ±8 g for the magnetometer, and ±2000 dps for the gyroscope. The sampling frequency of the measurement was set to be at least 30 Hz to secure a fast enough bandwidth for human postural movement, which is mostly in the range of 1–5 Hz (Table 1).

### 2.3. Design and Prototype of Smart Clothing

**Figure 3:**
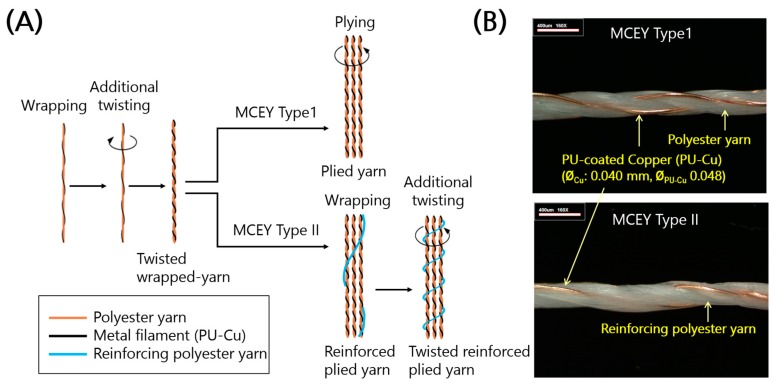
Development of conductive yarn. (**A**) Illustration of yarn manufacturing process for MCEY Types 1 and 2. (**B**) Lengthwise images of insulated MCEY (PU-Cu). Yarn Type 1 is a plied yarn of three strands of twisted wrapped yarn, and Yarn Type 2 is a twisted reinforced plied yarn of reinforcing polyester yarn wrapped around three strands of twisted wrapped yarn.

Two types of conductive yarn were developed for signal and power transmission lines to achieve wire-line networks between sensors and the coordinator (Figure 3). The two types were both metal composite embroidery yarn (MCEY) with different ratios of polyurethane-coated copper filament (PU-Cu) and polyester, and they were manufactured using different methods (Figure 3). Yarn Type 1 is a plied yarn of three strands of a twisted wrapped yarn of metal filament (PU-Cu) and polyester yarn. For enhanced yield strength, Yarn Type 2 is a twisted reinforced plied yarn of reinforcing polyester yarn wrapped around three strands of twisted wrapped yarn. The details of the yarn are explained in previous studies [5,14]. 

The linear density of the polyester yarn and its number of twists in the final manufacturing step were chosen to be 75 deniers (weight in grams per unit length of 9000 m) and 450 TM (twist per meter), respectively. The mechanical and electrical properties were examined to select the more reliable type of yarn type, including the electrical stability as a function of the stitch direction. The conductive yarn was embroidered for circuit networks on highly stretchable fabric to increase the stretchability and ensure that the activities of the wearer are not restricted. 

**Figure 4:**
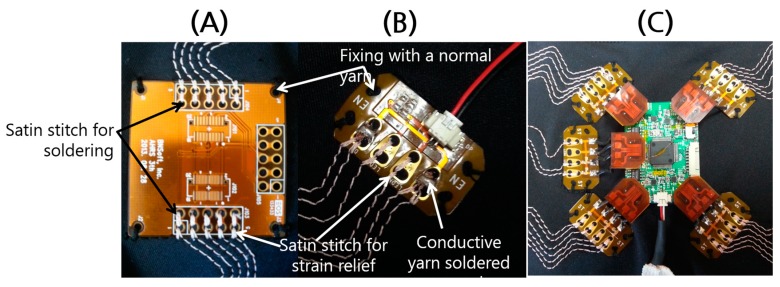
FPCBs for interconnection attached to clothes through a circuit patterning process using conductive yarn. (**A**) FPCB for sensor module. (**B**) FPCB for actuator. (**C**) Stretchable circuits.

A flexible printed circuit board (FPCB) was designed for interconnection to attach and detach the sensor module conveniently. The FPCB is connected to the embroidered circuit of the conductive yarn at a certain position on the clothes during a circuit pattering process on a computer numerical control (CNC) embroidery machine. To firmly fix the module to the clothes and provide strain relief for avoiding metal breakage of the solder connections, a pair of satin stitches was applied on the conductive yarn at a pair of holes on the FPCB for the interconnection (Figure 4).

A prototype of the smart clothing was constructed to combine the sensor modules, connecting networks, and interconnectors. For accurate measurement of motions, a basic pattern was designed to be in tight contact with the human body based on the pattern reduction method [15] using a highly stretchable knit fabric (89% polyamide, 11% polyurethane, g/m^2^). The cutting lines of the clothes were designed to secure the 3D wearable circuit network. The working orders were carefully designed for efficient and feasible processes of embroidery circuit patterning and garment sewing. The FPCB and embroidered circuit were connected with each other by soldering. The sensors were attached to the FPCBs for interconnection to complete the prototype.

### 2.4. Feasibility Test of Prototype for Posture Monitoring

Six healthy male subjects who had no history of spine or musculoskeletal disorder participated in the experiment. The average age, weight, and height of the subjects were 22.5 ± 2.5 years, 70.8 ± 9.9 kg, and 170.0 ± 4.8 cm, respectively. Prior to the data collection, the subjects read and signed an informed consent form approved by the institutional review board of KAIST.

The system was calibrated with the subject in a sitting posture with leaning in the anterior–posterior (AP) and medial–lateral (ML) directions, which led to pitch and roll rotations of the upper body, respectively. Subjects were initially sitting upright with their arms held at the chest. Next, they gradually leaned to one of the four directions and returned to an upright position around within 10 s, which was repeated for 10 trials for each direction. The magnitudes of the maximum lean to the AP and ML directions were 37.5° ± 9.3°, −27.6° ± 9.5°, 7.8° ± 3.0°, and −5.1° ± 2.9°, respectively. To monitor the sitting posture during daily activity, the subjects remained seated in front of a desk for an hour while using a desktop computer.

The angle of the torso was measured by both the sensor modules and an optical motion capture system (Hawk, Motion Analysis Corporation, Santa Rosa, CA, USA) with a sampling frequency of 30 Hz. The torso angle was calculated from the smart clothing using the average position data from the IMU modules at each shoulder and the waist. Four motion capture cameras were placed about 3 m behind the subjects, who had four markers arranged in a cross shape at the C7 vertebra and two markers at the IMU modules on both shoulders. The measurement data from the IMU modules and motion capture camera were filtered by a moving average filter with the last three data and a 5th-order Butterworth low-pass filter with a cut-off frequency of 1 Hz, respectively. The correlation between the torso angles measured by the camera and IMU modules were calculated by the goodness of fit in linear regression.

## 3. Results and Discussion

### 3.1. Mechanical and Electrical Properties of MCEY (PU-Cu)

The mechanical and electrical characteristics of the two types of conductive yarn are listed in Table 2. The yarn had very low resistance of around 0.05 Ω/cm, it is flexible and resistant to bending, and it can be embroidered on clothes to pattern various wearable circuits. By introducing reinforcing polyester (Yarn Type 2), the yield strength of the yarn was increased by 15% without significantly changing the electrical resistance (*p* = 0.12) (Table 2). When producing an embroidered circuit, considerable stress is imposed on the conductive yarn, and there is different stitching stress in the four different running directions of the CNC embroidery machine (from front to back (D1), from left to right (D2), from back to front (D3), and from right to left (D4)) [5]. Although the electrical resistance of embroidered circuits made from each yarn varied between different stitch directions, the embroidered circuits of Yarn Type 2 showed more consistent electrical stability (Table 3) due to its enhanced yield strength, which is desirable for stable connectivity of the embroidered circuit. Thus, Yarn Type 2 was chosen to make the prototype.

### 3.2. Calibration of the Prototype

**Figure 5:**
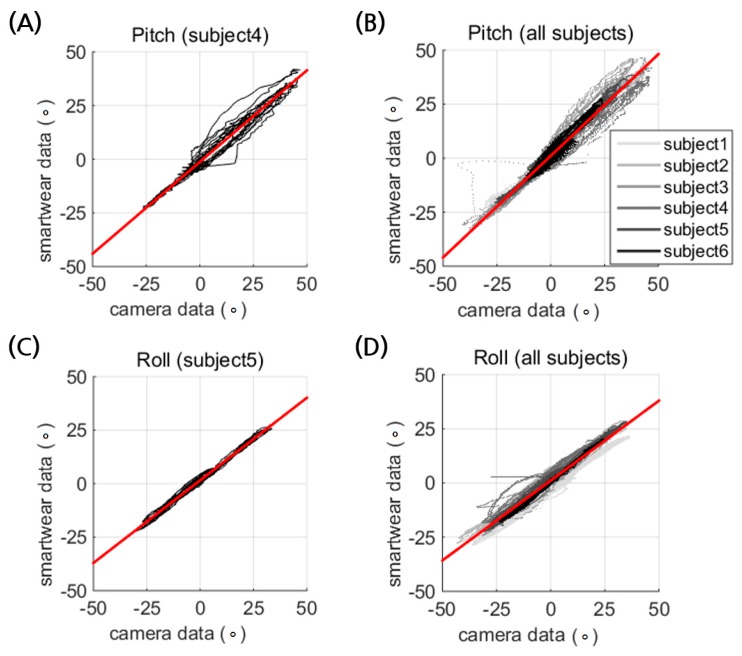
Calibration of the smart wear for (**A**,**B**), pitch and (**C**,**D**) roll directional tilt of the torso ranging from −40° to 40°. The results of one representative subject (**A**,**C**) are followed by data from 6 subjects (**B**,**D**), with solid lines in different shades of gray indicating different subjects. The linear regression of each data is depicted as a red line.

**Figure 6:**
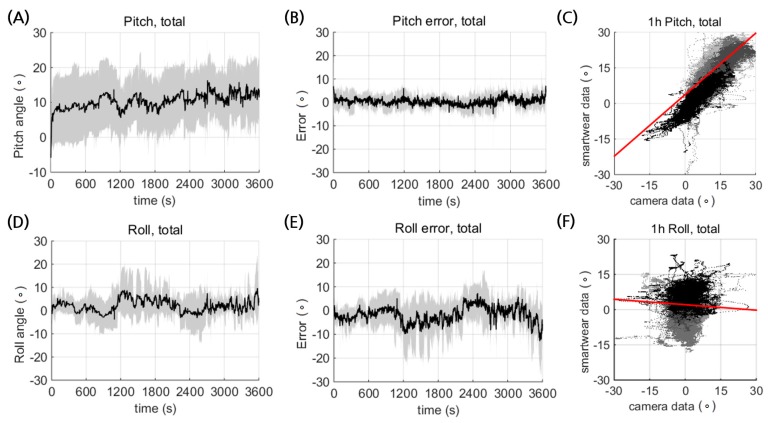
Postural estimation of pitch and roll from the smart wear during an hour of sitting activity. Average temporal trajectories of (**A**) pitch and (**D**) roll, and the corresponding temporal error profiles for (**B**) pitch and (**E**) roll. Mean values and standard deviation are shown as black solid lines and gray shading, respectively. Correlation between the estimated (**C**) pitch and (**F**) roll posture from the smart wear and the measurements from the camera. Different solid lines with different shades of gray indicate different subjects.

The prototype of the smart wear showed reasonably good estimation of the postural tilt of the torso with strong linearity in the pitch (R^2^ = 0.974) and roll directions (R^2^ = 0.976) (Figure 5, Table 4 and Table 5). The linearity coefficient of the pitch estimation is close to unity, but the IMU modules underestimate the roll rotation by an average factor of 0.751. The underestimated roll estimation is attributed to the rotational coupling of the roll tilt and the yaw rotation, which was observed when the subjects were asked to roll. The two IMU modules on the shoulders face upward and forward, which results in coupling of the roll rotation with yaw. The underestimation was related to the constant initial pitch angle, as shown in Figure 6D. Thus, it could be calibrated by using a coefficient. This implies that the motion coupling of the roll and yaw rotation of the body should be considered to write an estimation algorithm of posture from the IMU modules, which inherits the issue of relative coordination by being placed on the body.

### 3.3. System Verification under Free Movement

The prototype of the smart wear could estimate the posture reliably for durations over an hour (Figure 6). The temporal profile of the pitch and roll showed no significant low frequency drift by accumulated randomized error (Figure 6A,D). This implies that the smart wear could be reliably used for postural monitoring. The error profiles also showed no time dependency (Figure 6B,E), which indicates temporal reliability of the IMU measurements. 

There was no significant increase in the temperature of the IMU modules or the electrical circuits (data not shown). Although both the pitch and roll estimation showed high correlation with the camera data during calibration (Figure 5), the average estimation errors significantly increased during hour-long measurement (*p* ≪ 0.01; Table 6). Despite the smart wear being made with a tight stretchable texture, there may have been relative motion of the IMU modules and the body during the hour-long measurement, which would contribute to increasing the estimation error. 

**Figure 7:**
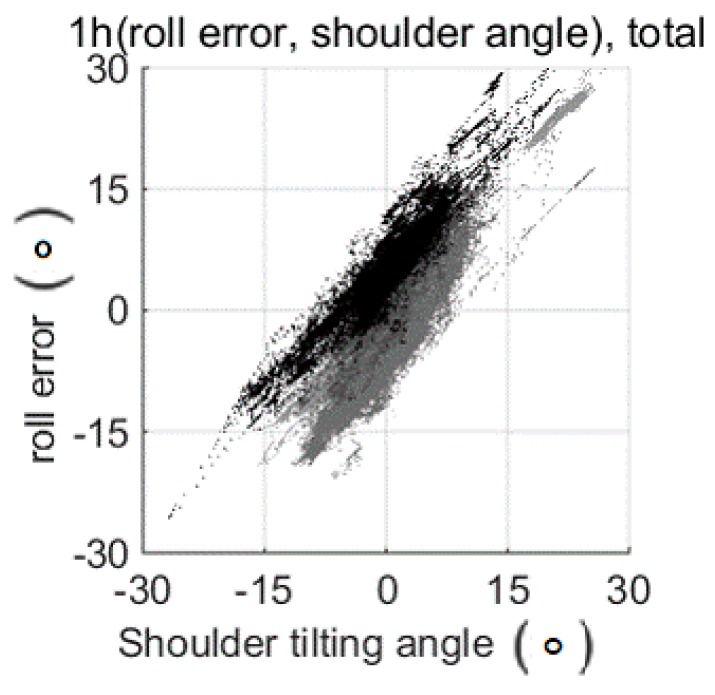
Correlation between the shoulder tilting angle from the spine and the roll estimation error. The shoulder angle and spine angle were measured by a camera, and the roll estimation error was calculated using the roll posture from the smart wear and the camera.

The deteriorated posture estimation appeared to be more significant for roll than pitch (Figure 6C,F; Table 6) due to the larger relative motion between the spine and the shoulder (Figure 7). The smart wear measured the spine angle by averaging two angle data from the IMU sensors on the shoulders. Thus, estimation error could occur due to the relative motion between the spine and the shoulders. For instance, the measured angle could change as the shoulder moves while the spine does not move. 

Figure 7 shows the relation between the shoulder tilting angle from the spine and the roll estimation error. For the pitch angle, the effect of the shoulder motion was small because it was difficult for the upper part of the shoulder and the spine to rotate separately. However, for the roll angle, the upper part of the shoulders could rotate separately, such as when shrinking up the shoulders, so large error occurred during the free movement experiment. During the sitting activity, the subjects used their arms and shoulders in various ways, which resulted in relative motion between the shoulders and the spine, leading to estimation error. The results imply that the proposed smart wear could monitor AP movement more reliably than movements in posture directions.

## 4. Conclusions

We developed smart clothing for postural monitoring and tested the feasibility of a prototype. To integrate the IMU modules, we developed conductive yarn with increased yield strength and used it to embroider an electrical circuit network. An FPCB was designed for interconnections to conveniently attach and detach the sensor module. The prototype was tested for monitoring sitting posture and showed reasonable estimates of the pitch and roll motion. The results show that the proposed smart clothing is feasible for postural monitoring. 

Auditory feedback could be added to the system to help prevent lower back pain among users. To improve the posture estimates, an algorithm should be developed to compensate for the coupling of body motion. Further studies will be carried out to develop encapsulation techniques to protect the sensor board and maintain durable interconnections.

## Figures and Tables

**Table 1:** Sensor characteristics of our system and other products.

	Range	Resolution	Sample Rate	Noise
**Our system**				
Accelerometer	±16 g	13 bits	6.25–3200 Hz	28.6 mg
Gyroscope	±2000 deg/s	14 bits	100–800 Hz	0.03 deg/s
Magnetometer	±8 Gauss	14 bits	0.75–75 Hz	2 mGauss
**APDM**				
Accelerometer	±16 g	14 bits	20–200 Hz	120 μg
Gyroscope	±2000 deg/s	16 bits	20–200 Hz	0.025 deg/s
Magnetometer	±8 Gauss	12 bits	20–200 Hz	2 mGauss
**Shimmer3**				
Accelerometer	±16 g	16 bits	512 Hz	2.80 mg
Gyroscope	±2000 deg/s	16 bits	512 Hz	0.048 deg/s
Magnetometer	±8.1 Gauss	16 bits	512 Hz	8 mGauss

**Table 2:** Characteristics of the metal composite embroidery yarn (MCEY).

Yarn Type	Composition (wt % of PU-Cu:Polyester)	Resistance (Ω/cm)	Linear Density (Denier)	Load at Yield (Offset 0.2%) (N)	Max. Load (N)	Strain (%)
1	57.3:42.7	0.052	549	3.92	13.3	13.1
2	50.3:49.7	0.051	634	4.48	17.4	15.2

**Table 3:** Electrical resistance (Ω/cm) of the MCEY embroidered circuit line (2.5 mm running stitches).

Yarn Type	D1 Front-to-Back	D2 Left-to-Right	D3 Back-to-Front	D4 Right-to-Left
1	0.766 ± 0.095	0.815 ± 0.017	0.727 ± 0.022	0.865 ± 0.039
2	0.966 ± 0.019	0.767 ± 0.015	0.726 ± 0.009	0.940 ± 0.019

**Table 4:** Pitch data relation: smart wear data = a × camera data + b, R^2^.

Subject	1	2	3	4	5	6	Average
a	0.924	0.963	1.01	0.854	0.964	1.03	0.958 ± 0.063
b	1.43	0.643	3.04	−1.31	1.72	0.871	1.07 ±1.44
R^2^	0.975	0.983	0.970	0.979	0.987	0.942	0.973 ± 0.016

**Table 5:** Pitch data relation: smart wear data = a × camera data + b, R^2^.

Subject	1	2	3	4	5	6	Average
a	0.721	0.723	0.763	0.727	0.772	0.808	0.752 ± 0.035
b	−2.81	2.96	0.974	3.59	1.54	0.375	1.10 ± 2.27
R^2^	0.987	0.985	0.975	0.902	0.992	0.996	0.973 ± 0.035

**Table 6:** RMS error in the calibration and one-hour experiment.

Subject		1	2	3	4	5	6	Average
Pitch RMSE	Cal.	2.07	2.02	3.40	2.74	1.78	2.11	2.35 ± 0.604
	1 h exp.	2.33	5.64	3.62	4.67	2.89	3.62	3.80 ± 1.20
Roll RMSE	Cal.	4.05	4.45	2.55	4.90	1.40	0.804	3.03 ± 1.70
	1 h exp.	2.07	6.36	6.72	15.2	4.98	6.76	7.02 ± 4.39
